# Calcium Supplementation, Risk of Cardiovascular Diseases, and Mortality: A Real-World Study of the Korean National Health Insurance Service Data

**DOI:** 10.3390/nu14122538

**Published:** 2022-06-18

**Authors:** Jae-Min Park, Bora Lee, Young-Sang Kim, Kyung-Won Hong, Yon Chul Park, Dong Hyeok Shin, Yonghwan Kim, Kunhee Han, Kwangyoon Kim, Junghwa Shin, Mina Kim, Bom-Taeck Kim

**Affiliations:** 1 Department of Family Medicine, College of Medicine, Gangnam Severance Hospital, Yonsei University, Seoul 06273, Korea; milkcandy@yuhs.ac; 2 Department of Medicine, Graduate School of Medicine, Yonsei University, Seoul 03722, Korea; 3 Institute of Health & Environment, Seoul National University, Seoul 08826, Korea; mintbora0125@gmail.com; 4 Department of Family Medicine, CHA Bundang Medical Centre, CHA University, Seongnam 13496, Korea; zeroup@cha.ac.kr; 5 Healthcare R&D Division, Theragen Bio Co., Ltd., Suwon 16229, Korea; kyungwon.hong@theragenbio.com; 6 Department of Family Medicine, Wonju Severance Christian Hospital, Wonju 26426, Korea; iamyonchul@yonsei.ac.kr; 7 Department of Family Medicine, Major Clinic, Seoul 06279, Korea; drsdh@hanmail.net; 8 Department of Family Medicine, Chungbuk National University Hospital, Cheongju 28644, Korea; airsantajin@gmail.com; 9 Department of Family Medicine, Seonam Hospital, Seoul 08049, Korea; hankawow@gmail.com; 10 Department of Family Practice and Community Health, Ajou University Hospital, Ajou University, Suwon 16499, Korea; hikari65@hanmail.net (K.K.); yooniregina@ajou.ac.kr (J.S.); 11 Data Science Team, Hanmi Pharm. Co., Ltd., Seoul 05545, Korea; mina.kim92@hanmi.co.kr

**Keywords:** calcium supplementation, acute myocardial infarction, ischemic stroke, cardiovascular diseases, osteoporosis

## Abstract

Few studies have investigated the effects of calcium supplementation on cardiovascular outcomes in individuals with low calcium intake in real-world settings. This study examined the association between calcium supplementation and cardiovascular outcomes in the Korean population in a real-world setting. This large retrospective cohort study included patients aged ≥45 years first prescribed calcium supplements in 2010. Age- and sex-matched controls were recruited among those who had no prescription for calcium supplements. Longitudinal data were collected on 31 December 2018. Kaplan–Meier estimation and Cox proportional hazard regression analysis were performed. The cumulative incidence of acute myocardial infarction, ischemic stroke, and death was significantly higher in the calcium supplementation group than in the control group (*p* < 0.05 by log-rank test). The calcium supplementation group had a significantly higher risk of myocardial infarction, ischemic stroke, and death than the control group. Compared to the control group, the hazard ratios (95% confidence intervals) of the incidence of myocardial infarction, stroke, and death in the supplementation group were 1.14 (1.03–1.27), 1.12 (1.05–1.20), and 1.40 (1.32–1.50), respectively, after adjusting for confounding variables. Considering the associated cardiovascular risk, calcium supplementation for osteoporosis treatment should be administered cautiously.

## 1. Introduction

Calcium is a macromineral that plays several essential biological roles in physiology and pathology, with its most universally known function being a crucial structural bone component [1,2]. The clinical guidelines proposed by the American Association of Clinical Endocrinologists, the American College of Endocrinology, the National Osteoporosis Foundation, and the National Osteoporosis Group recommend a daily calcium intake ranging between 700 and 1200 mg for adults aged ≥50 years [3,4,5].

Calcium supplementation has been advocated for treating and preventing osteoporosis [6]. However, studies have reported negative cardiovascular effects of calcium supplement intake and stated that calcium intake might be related to an increased risk of myocardial infarction [7] and ischemic stroke [8]. Although a previous meta-analysis by Lewis et al. showed no relationship between calcium supplementation and cardiovascular event risk [9], a recent meta-analysis of 13 randomized, double-blind, placebo-controlled trials showed that calcium supplementation increased the risk of cardiovascular diseases [10]. However, most studies were conducted in Western countries with high calcium intake, and evidence regarding populations with low calcium intake is rare. It is necessary to study the effects of calcium supplementation on cardiovascular outcomes in these populations as Asian populations tend to have relatively low dietary calcium intake, which is only one-third to one-fifth of that in Western populations [11].

Koreans are known to have little calcium intake, and the daily calcium intake for adults aged ≥50 years is 470 mg [12]. Thus, in clinical practice, several Korean physicians prescribe calcium supplementation to patients for the prevention and treatment of osteoporosis, especially in postmenopausal women and elderly men. However, few studies have investigated the effects of calcium supplementation on cardiovascular outcomes in individuals with low calcium intake. Moreover, few studies have examined the relationship between calcium supplementation and cardiovascular outcomes in real-world settings. This study aimed to examine the association between calcium supplementation and cardiovascular outcomes in the Korean population in a real-world setting. We conducted a large retrospective cohort study using the National Health Insurance Service (NHIS) cohort, which included the entire population of South Korea.

## 2. Materials and Methods

### 2.1. Data Source

This study was based on mandatory health insurance claims from the NHIS claims database from 1 January 2007 to 31 December 2018. The NHIS, run by the government of South Korea, covers the entire population of South Korea (i.e., approximately 52 million people) [13]. All healthcare providers are required to submit all treatment data to the NHIS for insurance reimbursement. The NHIS database contains enormous epidemiological and medical service information, including demographics, diagnoses, and prescription records (drug code and days prescribed). The diagnosis codes are standardized in accordance with the 7th version of the Korean Classification of Disease, which is a modified version of the 10th edition of the International Classification of Diseases. The database also includes health check-up data with questionnaires on health-related lifestyles and behaviors, collected through a national health examination every other year from office workers aged >40 years and once a year from non-office workers. After identification, we used the NHIS database.

The study protocol conformed to the ethical guidelines of the 1975 Declaration of Helsinki, as reflected in the a priori approval by the Institutional Review Board of the Ajou University Hospital (approval number: AJIRB-SBR-EXP-19-425). The requirement to acquire informed consent from participants was waived because this was a retrospective observational study with an anonymized dataset.

### 2.2. Study Population and Design

**Figure 1:**
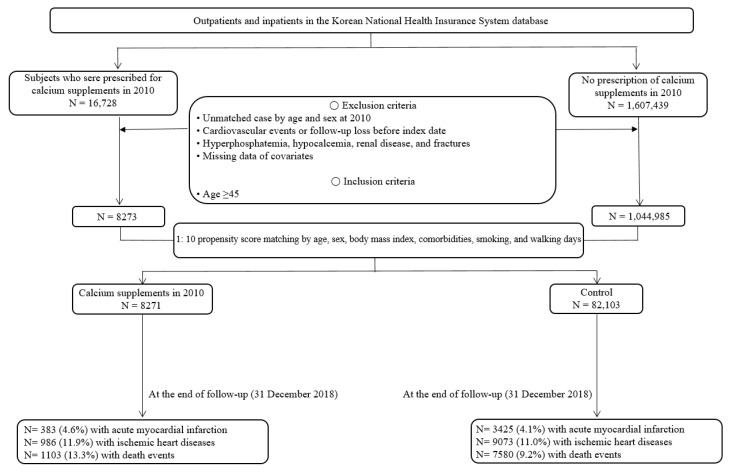
Flowchart of participant selection from the National Health Insurance Service database of Korea.

This was a retrospective cohort study based on the NHIS database. Patients aged ≥45 years who were first prescribed calcium supplements in 2010 were included. The date of the first prescription was defined as the index date. Among those who had no prescription for calcium supplements, participants of the same age and sex as those in the prescription group were enrolled as controls. Longitudinal data were collected on 31 December 2018. A schematic representation of this study is shown in Figure 1. We identified 16,728 participants who were newly prescribed calcium supplements in 2010 as cases and 1,607,439 participants who had no prescription for calcium supplements as controls. Among them, we excluded those with unmatched age and sex, cardiovascular events before the index date, loss of follow-up before the index date, hyperphosphatemia, hypocalcemia, renal disease, fractures, or missing covariates. We included participants who were aged >45 years. After exclusion and inclusion, 8273 patients and 1,044,985 controls were included. After a 1:10 propensity score matching, 8271 cases and 82,103 controls were enrolled in the main analyses.

### 2.3. Exposures

The primary drug exposure of interest was the intake and dosage of calcium supplements. Prescribed data, including the drug code and duration for calcium supplements, were collected to calculate the cumulative defined daily dose according to the ingredient matched to the Anatomical Therapeutic Classification of drugs [14]. Individuals were divided into two groups: the calcium supplementation (at least one prescription for calcium supplements in 2010) and control (no history of calcium supplements in 2002–2018) groups.

### 2.4. Outcomes

The primary outcome was the first occurrence of a cardiovascular event, including acute myocardial infarction and ischemic stroke. The follow-up duration was computed as the period between the index and the event or censored dates. The event date was defined as the first date of an I21 code diagnosis of acute myocardial infarction or an I63 code diagnosis of ischemic stroke, whichever occurred first. Combined cardiovascular events were defined as myocardial infarction or ischemic stroke. All causes of death were defined based on information on death, and its date, for all participants in this cohort. The censored date was defined as the date recorded in the death registry or at the end of the study (31 December 2018).

### 2.5. Covariates

Demographic data included age and sex. Age was measured on an index date. From the health examination data recorded closest to the index date, we collected data on BMI and self-reported health-related behaviors, including smoking status (never smoked, ex-smoker, or current smoker) and frequency of walking days per week. The Charlson Comorbidity Index (CCI) was computed based on the ICD-10 codes in the medical database using the weighted sum of comorbidities [15].

### 2.6. Statistical Analyses

Descriptive statistics were reported as mean and standard deviation for continuous variables and frequency and percentages for categorical variables. Statistical differences between groups were evaluated using the Student’s *t*-test or Wilcoxon’s rank-sum test for continuous variables and chi-squared test or Fisher’s exact test for categorical variables, as appropriate, after assumption testing. A propensity score (PS) matching analysis was performed to minimize the probability of selection bias between the calcium supplementation and control groups. The PS was generated using multiple logistic regression models, including age, sex, BMI, CCI, smoking status, walking days per week, osteoporosis, and dyslipidemia. We used the nearest available matching (1:10) method to estimate the PS using a caliper of 0.15. Balance was assessed by a standardized mean difference of <0.1 after matching. Based on the matched data, Kaplan–Meier estimation was used to calculate the cumulative incidence of the primary outcome. A Cox proportional hazard regression analysis was used to assess the effect of calcium supplementation on cardiovascular events or death. The proportional hazards assumption of the model was valid for each event based on the graphical and statistical test using the scaled Schoenfeld residuals (*p* < 0.1) [16]. Except for the proportional hazard assumption test, a two-tailed *p*-value < 0.05 was considered statistically significant. All analyses were performed using SAS version 9.4 (SAS Institute, Cary, NC, USA) and R version 3.4.3 (R Foundation for Statistical Computing, Vienna, Austria).

## 3. Results

Table 1 presents the baseline characteristics of the study participants according to their calcium supplementation prescription before and after PS matching. Comparing the standardized mean difference between individual covariates in the calcium supplement and control groups shows a good covariate balance (standardized mean difference < 0.1). Of the total matched group, males comprised 21.4% (*n* = 1767) of the calcium supplementation group and 18.8% (*n* = 15,423) of the control group. The mean age, BMI, and smoking status were 61.5 ± 10.3 years, 23.9 ± 3.3 kg/m^2^, and 9.4% (*n* = 778) in the calcium supplementation group and 62.0 ± 9.2 years, 24.0 ± 3.2 kg/m^2^, and 9.1% (*n* = 7479) in the control group, respectively. The Charlson Comorbidity Index was 3.2 ± 1.7 and the average walking days per week were 2.5 ± 2.6 days in both the calcium supplementation and control groups. Patients with osteoporosis and dyslipidemia were 67.5% and 45.2% in the calcium supplementation group and 65.0% and 43.0% in the control group, respectively. The median follow-up time was 6.76 years. During the follow-up period, 4.6% and 4.1% of acute myocardial infarction cases, 11.9% and 11.0% of ischemic stroke cases, and 13.3% and 9.2% of death events were identified in the calcium supplement and control groups, respectively (Figure 1).

**Figure 2:**
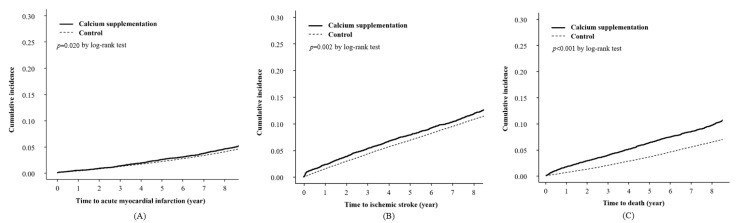
Kaplan–Meier curves with the cumulative incidence of acute myocardial infarction (**A**), ischemic stroke (**B**), and death events (**C**) following the prescription of calcium supplements.

We examined whether the incidence rates of cardiovascular events or death differed according to calcium supplement prescriptions (Figure 2). During the observational period, the cumulative incidence of cardiovascular events, including acute myocardial infarction and ischemic stroke, was significantly higher in the calcium supplementation group than in the control group (*p* < 0.05 by log-rank test; Figure 2A,B). Moreover, the cumulative incidence of death was significantly higher in the calcium supplementation group than in the control group (*p* < 0.001 by log-rank test; Figure 2C).

Table 2 shows the hazard ratio (HR) for cardiovascular events according to the prescription of calcium supplementation determined through a Cox regression analysis. The calcium supplementation group had a significantly higher risk of myocardial infarction and ischemic stroke than the control group even though the covariates were included sequentially. Compared to the control group, the HRs (95% confidence interval [CIs]) for the incidence of myocardial infarction and stroke in the supplementation group were 1.24 (1.03–1.27) and 1.12 (1.05–1.20), respectively, after adjusting for age, sex, BMI, comorbidities, smoking, walking days per week, osteoporosis, and dyslipidemia. Moreover, compared to the control group, the HR (95% CIs) for the incidence of death events in the supplementation group was 1.47 (1.40–1.54) after adjusting for the same confounding variables.

Table 3 presents the Cox proportional hazards model for cardiovascular events. While age, male sex, BMI, comorbidities, and current smoking status were associated with an increased risk of myocardial infarction and ischemic stroke, walking days per week was associated with a decreased risk.

## 4. Discussion

In this large-scale nationwide cohort study, the cumulative incidence and HR for acute myocardial infarction, ischemic stroke, and death events were higher in the calcium supplementation group than in the control group in a Korean population after adjusting for confounding variables. Calcium supplementation was significantly associated with an increased risk of acute myocardial infarction, ischemic stroke, and death in the Korean population. Studies linking calcium supplementation with cardiovascular outcomes and mortality have reported inconsistent results. While two previous meta-analyses by Bolland et al. [7,17] reported an association between calcium supplementation and the risk of myocardial infarction, two other meta-analyses by Chung et al. [18] and Lewis et al. [9] showed no relationship between calcium supplementation and cardiovascular disease risk. However, a recent meta-analysis by Myung et al. showed that calcium supplementation increased the risk of coronary heart disease [10]. Abajo et al. suggested that calcium supplementation might increase the risk of ischemic stroke [8]. Furthermore, Michaëlsson et al. found that a high calcium intake is related to high all-cause mortality [19]. Our findings are consistent with those of previous studies which showed that calcium supplementation is associated with an increased risk of myocardial infarction [7,10,17], ischemic stroke [8], and death [19] in other populations. Moreover, our findings suggest that calcium supplementation may increase the risk of myocardial infarction and ischemic stroke in Koreans, who are known to have little calcium intake. Although previous studies in Western countries with high calcium intakes investigated the association between calcium supplementation and cardiovascular outcomes, evidence regarding a population with low calcium intake is scarce. In addition, few studies have examined the effects of calcium supplementation on cardiovascular outcomes by using real-world data. This is the first large population-based cohort study to use real-world data to reveal a close relationship between calcium supplementation and the incidence of myocardial infarction and ischemic stroke. Thus, our results confirm earlier findings regarding the association between calcium supplementation and cardiovascular outcomes.

Cardiovascular disease and osteoporosis are major causes of mortality and morbidity worldwide, particularly among older adults in developed countries. They share risk factors. Moreover, growing evidence shows they share pathogenic mechanisms, including inflammation and imbalance in mineral metabolism and the renin–angiotensin–aldosterone system [20,21]. Studies have indicated a relationship between cardiovascular disease and low bone mass [22,23,24]. In this study, osteoporosis was matched using PS and adjusted for in the Cox regression analysis to minimize the potential confounding effect of osteoporosis on cardiovascular events. There were few studies controlled for the diagnosis of osteoporosis investigating the association between calcium supplementation and cardiovascular events. Therefore, our findings expand on earlier findings regarding the relationship between calcium supplementation and cardiovascular outcomes.

Several possible mechanisms can be attributed to the significantly increased risk of myocardial infarction and ischemic stroke through calcium supplementation. Although dietary calcium has smaller effects on circulating calcium concentration, calcium supplementation could abruptly increase serum calcium concentration [25,26,27]. An intermittent acute rise in circulating calcium concentration maintained over long periods would eventually promote vascular calcification, which is a significant marker of atherosclerosis [28,29]. Furthermore, elevated serum calcium elevates blood pressure, possibly mediated by extracellular and intracellular calcium changes, altering the renin–angiotensin–aldosterone system and regulating vascular smooth muscle contraction [30,31,32].

Direct vascular effects are not the only possible mechanism to be considered. Calcium participates in the coagulation pathway and controls platelet function through calcium-sensing receptors. Platelets, which play a key role in the initiation of coagulation, express calcium-sensing receptors [33], indicating that extracellular calcium concentration can affect their activity. Platelet activation is higher in individuals with primary hyperparathyroidism before surgical cure than in the same group after surgical cure, suggesting that increased serum calcium levels might directly activate platelets [34]. Moreover, this is sustained by increased blood coagulability 4 h after an oral calcium supplement load in postmenopausal women [32].

In this study, ischemic stroke occurred faster in the calcium supplementation group compared with that in the control group, and this gap was maintained for the rest of study period. We thought that this could act as evidence of the short-term risk of calcium supplementation on the incidence of ischemic stroke. This means that calcium supplement intake affects the risk of ischemic stroke over short periods; though after that, the incidence of ischemic stroke depends on the age of participants. That could be a reason why the gap between the two groups persisted for the rest of the follow-up period in this study.

This study has several limitations. First, it was based on the medical insurance claims database (NHIS database). Since insurance claims databases provide limited information on patients and do not include entire medical records, the diagnostic accuracy of myocardial infarction and ischemic stroke cannot be guaranteed, and risks and events of myocardial infarction and ischemic stroke could have been overestimated. Second, we could not report on the dose of calcium and vitamin D supplementations due to dataset limitation, although these doses are important issues. Further research is warranted to examine the relationship between calcium supplementation and cardiovascular outcomes, considering calcium dose and vitamin D supplementation in a real-world setting. Third, people can also purchase and take calcium supplements as over-the-counter medicines without a doctor’s prescription. However, we could not consider over-the-counter calcium supplementation in the analysis since this study could not determine whether each participant took such supplements. Further research that considers over-the-counter calcium supplementation is needed. Fourth, this study did not consider hormone replacement therapy in women, which may affect osteoporosis and cardiovascular events [35,36]. However, we could not determine whether each participant was treated with hormone replacement therapy. Further studies are warranted to explicate the association between calcium supplementation and cardiovascular events by confirming whether each female participant was treated with hormone replacement therapy. Fifth, since those with calcium supplement use may have had a prior low dietary calcium intake, dietary calcium intake is an important confounding factor. However, since information on participants’ dietary calcium intake was not included in the NHIS claims dataset, we could not determine whether the participants had a low calcium intake. Further studies that consider participants’ dietary calcium intake in a real-world setting are needed. Lastly, since this was a retrospective observational study based on the claims database, not a randomized controlled trial, it is possible that residual confounding factors remain. Despite these potential limitations, we conducted a large population-based cohort study with a relatively long duration using real-world data from the entire population of South Korea. Moreover, to minimize confounding factors, age, sex, BMI, smoking, and well-known cardiovascular comorbidities were matched using PS and adjusted for in the Cox regression analysis.

## 5. Conclusions

In this real-world, large-scale cohort study, the cumulative incidence and HR of acute myocardial infarction, ischemic stroke, and death increased with calcium supplementation compared with those observed without supplementation after adjusting for potential confounding variables. Considering the associated cardiovascular risk, calcium supplementation for osteoporosis treatment should be administered cautiously.

## Figures and Tables

**Table 1:** Baseline characteristics of study participants according to calcium supplement prescription before and after propensity score matching.

	Calcium Supplements (*n* = 8273)	Control (*n* = 1,044,985)	SMD	Calcium Supplements (*n* = 8271)	Control (*n* = 82,103)	SMD
Age (years)	61.5 ± 10.3	59.7 ± 9.1	0.198	61.5 ± 10.3	62.0 ± 9.2	0.054
Sex			0.402			0.090
Male	1769 (21.4)	121,516 (11.6%)		1767 (21.4)	15,423 (18.8)	
Female	8522 (78.6)	923,469 (88.4%)		6504 (78.6)	66,680 (81.2)	
BMI (kg/m^2^)	23.9 ± 3.3	24.0 ± 3.2	0.031	23.9 ± 3.3	24.0 ± 3.2	0.031
Charlson Comorbidity Index	3.2 ± 1.7	1.7 ± 1.6	0.937	3.2 ± 1.7	3.2 ± 1.7	<0.001
Smoking						
Never smoker	6885 (83.2)	923,266 (88.4)		6884 (83.2)	68,301 (83.2)	
Former smoker	609 (7.4)	50,429 (4.8)	0.254	609 (7.4)	6323 (7.7)	0.024
Current smoker	779 (9.4)	71,290 (6.8)	0.194	778 (9.4)	7479 (9.1)	0.020
Walking days per week (days)	2.5 ± 2.6	2.7 ± 2.6	0.077	2.5 ± 2.6	2.5 ± 2.6	<0.001
Osteoporosis	5586 (67.5)	210,985 (20.2)	1.160	5584 (67.5)	53,367 (65.0)	0.062
Dyslipidemia	3744 (45.3)	327,472 (31.3)	0.330	3742 (45.2)	35,305 (43.0)	0.049

Data are presented as mean ± standard deviation for continuous variables or *n* (percentage) for categorical variables. SMD, standardized mean difference; BMI, body mass index.

**Table 2:** Hazard ratios for cardiovascular events according to calcium supplementation prescription as determined using a Cox regression analysis.

	Myocardial Infarction	Ischemic Stroke	Death Events
Model 1	1.13 (1.02–1.26)	1.11 (1.04–1.18)	1.45 (1.36–1.54)
Model 2	1.14 (1.03–1.26)	1.11 (1.04–1.17)	1.38 (1.29–1.47)
Model 3	1.14 (1.03–1.27)	1.12 (1.05–1.20)	1.40 (1.32–1.50)

Model 1: Unadjusted. Model 2: Adjusted for age and sex. Model 3: Adjusted for age, sex, body mass index, comorbidities, smoking, walking days per week, osteoporosis, and dyslipidemia.

**Table 3:** Cox proportional hazards model for cardiovascular events.

	Myocardial Infarction		Ischemic Stroke	
	Crude HR (95% CI)	Adjusted HR (95% CI)	Crude HR (95% CI)	Adjusted HR (95% CI)
Group				
Control	1 (Reference)	1 (Reference)	1 (Reference)	1 (Reference)
Calcium supplementation	1.13 (1.02–1.26)	1.14 (1.03–1.27)	1.11 (1.04–1.18)	1.12 (1.05–1.20)
Age (year)	1.05 (1.04–1.05)	1.05 (1.04–1.04)	1.07 (1.07–1.08)	1.07 (1.06–1.07)
Sex				
Female	1 (Reference)	1 (Reference)	1 (Reference)	1 (Reference)
Male	1.36 (1.26–1.47)	1.33 (1.20–1.48)	1.21 (1.15–1.27)	1.17 (1.10–1.25)
BMI (kg/m^2^)	1.01 (1.01–1.02)	1.01 (1.01–1.02)	1.08 (1.08–1.09)	1.06 (1.06–1.07)
Charlson Comorbidity Index	1.17 (1.15–1.19)	1.18 (1.15–1.21)	1.22 (1.21–1.24)	1.17 (1.15–1.18)
Smoking				
Never smoker	1 (Reference)	1 (Reference)	1 (Reference)	1 (Reference)
Former smoker	1.11 (0.97–1.26)	0.92 (0.79–1.97)	1.09 (1.01–1.18)	0.97(0.88–1.06)
Current smoker	1.42 (1.28–1.57)	1.10 (1.01–1.19)	1.08 (1.01–1.16)	1.25 (1.26–1.35)
Walking days per week (days)	0.97 (0.96–0.98)	0.80 (0.74–0.87)	0.98 (0.97–0.98)	0.98 (0.98–0.99)
Osteoporosis				
No	1 (Reference)	1 (Reference)	1 (Reference)	1 (Reference)
Yes	1.33 (1.26–1.40)	1.05 (0.96–1.14)	1.58 (1.50–1.66)	1.03 (0.97–1.09)
Dyslipidemia				
No	1 (Reference)	1 (Reference)	1 (Reference)	1 (Reference)
Yes	1.10 (1.05–1.15)	1.08 (0.97–1.19)	1.29 (1.24–1.34)	1.02 (0.97–1.08)

Adjusted HRs were adjusted for calcium supplementation, age, sex, BMI, comorbidities, smoking, walking days per week, osteoporosis, and dyslipidemia. BMI, body mass index; HR, hazard ratio; CI, confidence interval.

## Data Availability

The data sets are available through approval and oversight by the Korean National Health Insurance Service.
